# SARS-CoV-2 excretion kinetics in nasopharyngeal and stool samples from the pediatric population

**DOI:** 10.3389/fmed.2023.1226207

**Published:** 2023-10-30

**Authors:** Haifa Khemiri, Mariem Gdoura, Samar Ben Halima, Henda Krichen, Cesare Cammà, Alessio Lorusso, Massimo Ancora, Adriano Di Pasquale, Asma Cherni, Henda Touzi, Amel Sadraoui, Zina Meddeb, Nahed Hogga, Radhia Ammi, Henda Triki, Sondes Haddad-Boubaker

**Affiliations:** ^1^Laboratory of Clinical Virology, WHO Regional Reference Laboratory for Poliomyelitis and Measles for the EMR, Institut Pasteur de Tunis, University of Tunis El Manar, Tunis, Tunisia; ^2^LR 20 IPT 02 Laboratory of Virus, Host and Vectors, Institut Pasteur de Tunis, University of Tunis El Manar, Tunis, Tunisia; ^3^Istituto Zooprofilattico Sperimentale dell'Abruzzo e del Molise, Teramo, Italy; ^4^Service of External Consultants, Institut Pasteur de Tunis, Tunis, Tunisia

**Keywords:** stool, pediatric population, infectious, variants, symptomatic, asymptomatic, SARS-CoV-2

## Abstract

**Background:**

The severe acute respiratory syndrome coronavirus 2 (SARS-CoV-2) is responsible for serious respiratory infections in humans. Even in the absence of respiratory symptoms, gastrointestinal (GI) signs were commonly reported in adults and children. Thus, oral–fecal transmission was suspected as a possible route of infection. The objective of this study was to describe RNA shedding in nasopharyngeal and stool samples obtained from asymptomatic and symptomatic children and to investigate virus viability.

**Methods:**

This study included 179 stool and 191 nasopharyngeal samples obtained from 71 children, which included symptomatic (*n* = 64) and asymptomatic (*n* = 7) ones. They were collected every 7 days from the onset of the infection until negativation. Viral RNA was detected by real-time RT-PCR, targeting the N and ORF1 genes. Whole-genome sequencing was performed for positive cases. Viral isolation was assessed on Vero cells, followed by molecular detection confirmation.

**Results:**

All cases included in this study (*n* = 71) were positive in their nasopharyngeal samples. SARS-CoV-2 RNA was detected in 36 stool samples obtained from 15 out of 71 (21.1%) children; 13 were symptomatic and two were asymptomatic. Excretion periods varied from 7 to 21 days and 7 to 14 days in nasopharyngeal and fecal samples, respectively. Four variants were detected: Alpha (*n* = 3), B.1.160 (*n* = 3), Delta (*n* = 7), and Omicron (*n* = 1). Inoculation of stool samples on cell culture showed no specific cytopathic effect. All cell culture supernatants were negative for RT-qPCR.

**Conclusion:**

Our study demonstrated nasopharyngeal and fecal shedding of SARS-CoV-2 RNA by children up to 21 and 14 days, respectively. Fecal shedding was recorded in symptomatic and asymptomatic children. Nevertheless, SARS-CoV-2 was not isolated from positive stool samples.

## 1. Introduction

At the beginning of the coronavirus disease 2019 (COVID-19) pandemic, available data suggested that the severe acute respiratory syndrome coronavirus 2 virus (SARS-CoV-2) was able to affect adults more than children. Up to May 2020, pediatric infections were limited to 1–5% of total recorded cases (1–4). Rapidly, several waves of COVID-19 have occurred around the world as a consequence of the emergence of multiple variants of concern (VOCs) (5–9). An increase in the number of pediatric cases was recorded, especially with the emergence of the Delta and Omicron variants (4, 10–14).

COVID-19 is mainly characterized by severe upper and lower respiratory tract infections in humans (15–19). Pediatric disease was, in general, less severe; most of the cases were asymptomatic or developed mild signs (14). Nevertheless, severe cases were also reported, requiring hospitalization and intensive care unit admission (4, 14, 20–22). Gastrointestinal (GI) signs were frequently reported, even in the absence of respiratory symptoms (3), estimated between 2 and 79% of cases according to different studies (3, 23–26). Diarrhea, vomiting, and nausea were estimated at 8.8–49.5%, 4.2–15.9%, and 4.2–29.4% of cases, respectively (26–33). Indeed, SARS-CoV-2 S protein binding to the host cell angiotensin-converting enzyme 2 (ACE2) receptor mediates viral entry. Although ACE2 is present throughout the respiratory tract (34), its expression is relatively low compared to the gastrointestinal tract, kidney, and myocardium (35). The most common GI symptom was diarrhea, which was generally noticed during the first 8 days of the infection (33, 36). Regarding child infection, GI was described as having a higher incidence, especially diarrhea and vomiting, estimated at 8–35.6% and 6.5–66.7%, respectively (33, 37–40). Shedding of SARS-CoV-2 RNA in stool samples was reported (3, 19, 41, 42). Thus, fecal–oral transmission was considered a possible route for SARS-CoV-2 transmission (41). Nevertheless, little is known about the virus shedding according to different variants and the duration of excretion, especially in pediatric patients and in symptomatic and asymptomatic cases. Furthermore, data on virus viability in positive stool samples using real-time PCR (RT-qPCR) are controversial (43). Several studies proved the absence of the live SARS-CoV-2 virus in feces (44–47), while two studies reported the possible presence of an infectious virus (48, 49).

This study aimed to investigate the excretion of SARS-CoV-2 RNA in nasopharyngeal and stool samples obtained from symptomatic and asymptomatic COVID-19 pediatric cases. Additionally, the virus viability of positive stool samples was explored by cell culture and confirmed by specific molecular detection applied to the cell culture product.

## 2. Materials and methods

### 2.1. Ethics statement

This study was approved by the Medical Ethics Committee of the Bechir Hamza Children’s Hospital of Tunis, Tunisia, under the reference “12/2021.” It was performed in accordance with the ethical standards of the 1964 Declaration of Helsinki and its later amendments, or comparable ethical standards. Written consent was obtained from their parents or their legal tutors.

### 2.2. Studied samples

**Figure 1:**
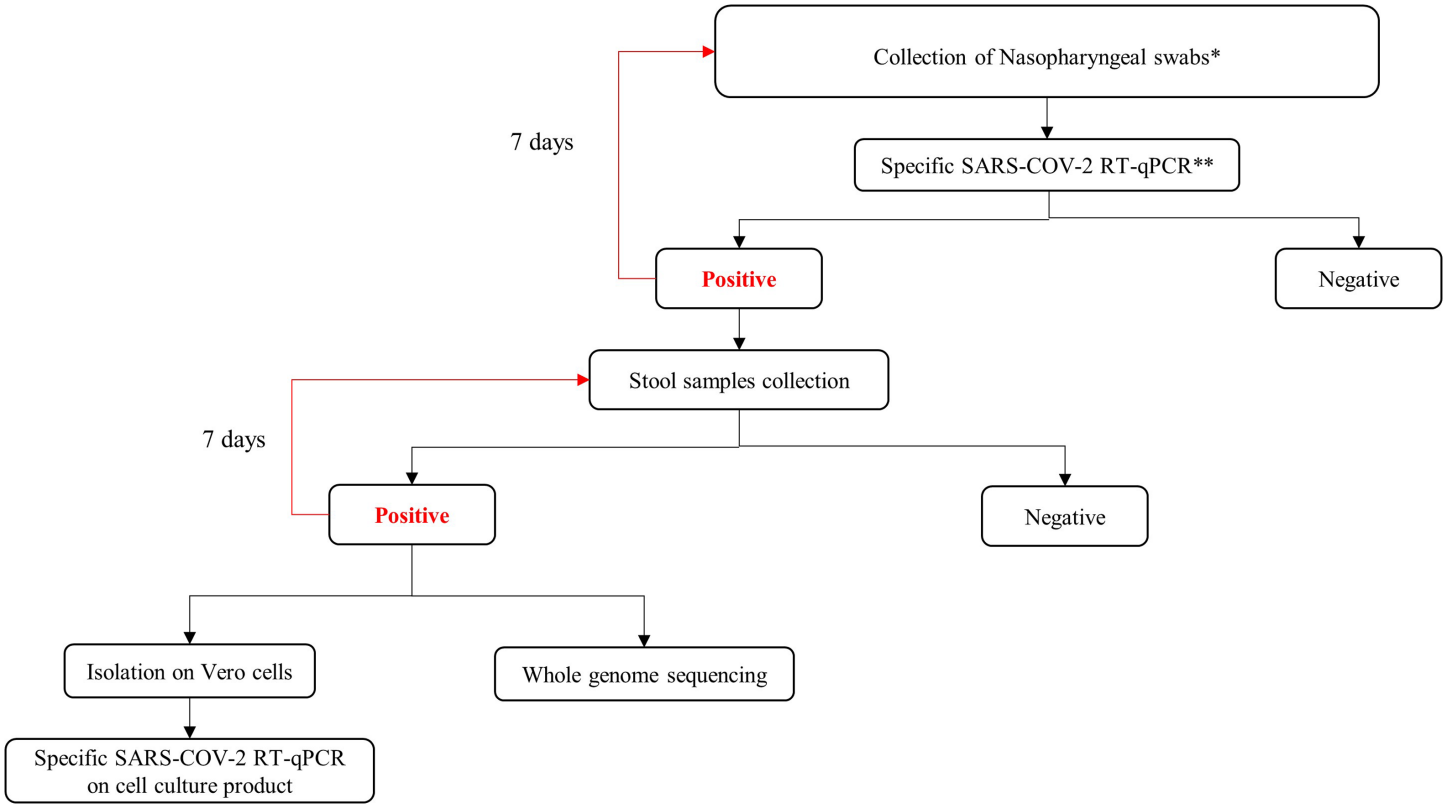
Workflow presenting the methodology used in this study. *Sample were collected from suspected COVID-19 pediatric cases and pediatric contact of confirmed adult cases; **WHO recommended protocol.

A total of 179 stool samples and 191 nasopharyngeal swabs were collected from 71 children between February 2021 and January 2022 at the Pasteur Institute of Tunis, in a pandemic context and after the obtention of their parents or their legal tutors’ consent. Details of each collected sample are listed in Supplementary Table 1. The study included 28 boys and 43 girls, with a sex ratio equivalent to 0.65. Their age ranged from 1 month to 18 years old, with a median age of 15 years. Patients who tested positive for SARS-CoV-2 RNA in the nasopharyngeal swab further underwent stool sample collection from these patients; nasopharyngeal swabs and stools were collected every 7 days until negative results were obtained (Figure 1). Two groups were considered. Group 1 included 160 stool samples collected from COVID-19 symptomatic children (*n* = 64), and Group 2 included 19 stool samples collected from COVID-19 asymptomatic children (*n* = 7), among the contacts of symptomatic cases (Table 1). According to the WHO Living Guidance for Clinical Management of COVID-19, symptomatic cases were defined as patients with mild, moderate, severe, or critical signs of COVID-19. Asymptomatic cases were defined as COVID-19 patients, confirmed by SARS-CoV-2 real-time PCR on nasopharyngeal samples with no signs or symptoms of an illness or disease (50).

**Table 1:** Characteristics of studied samples obtained from symptomatic and asymptomatic children.

Groups	Number of patients	Number of stool samples	Number of nasopharyngeal swabs
Group 1: symptomatic children	64	160	171
Group 2: asymptomatic children	7	19	20
Total	71	179	191

### 2.3. Nucleic acid extraction and detection by PCR

Nasopharyngeal and stool samples were processed in accordance with recommended good laboratory practices. Stool samples were treated with PBS/chloroform (1%) and centrifuged at 2500× *g* for 30 min, according to the WHO protocol for stool sample treatment (51–53). Viral RNA was extracted from 140 μL of the supernatants of nasopharyngeal and stool samples using the QIAamp Viral RNA Mini Kit (Qiagen, Hilden, Germany), according to the manufacturer’s instructions (54). The presence of SARS-CoV-2 RNA was determined with RT-qPCR using HKU (55) and IPT2-IPT4 protocols (Institut Pasteur, Paris) (56) as previously described (57) (Figure 1).

### 2.4. Whole-genome sequencing (WGS)

The whole genome of SARS-CoV-2 was obtained by next-generation sequencing using the COVIDSeq Test (Illumina Inc., San Diego, CA, USA) as previously described (58, 59) (Figure 1). The library preparation process used validated protocols at the “National Reference Centre for Whole-Genome Sequencing of microbial pathogens at Istituto Zooprofilattico Sperimentale dell’Abruzzo e del Molise IZSAM,” with the Hamilton Microlab STAR Liquid Handling System (Hamilton Robotics, Reno, NV, USA). NGS sequencing was achieved with the NextSeq 1,000 (Illumina Inc., San Diego, CA, USA), which provided read length data of 2 × 150 bp.

### 2.5. SARS-CoV-2 variant identification

Data analysis was automatically performed at the end of the sequencing run using the GENPAT platform at IZSAM in Teramo, as described in Molini et al. (59) and Di Pasquale et al. (60). Mapping to the Wuhan-Hu-1 reference genome (accession number NC_045512) was performed with the BWA tool (61), after quality control and trimming of the reads using FastQC and Trimmomatic (62). The consensus sequence was obtained using the iVar tool (63). The identification of SARS-CoV-2 lineage and sub-lineage was performed with the Pangolin (64) and Nextclade tools via the web^1^^,^ ^2^ (65). Boxplot was used to visualize our results (66).

### 2.6. Statistical analysis

A chi-square test was performed using R software (67), which evaluated whether there was a significant association between symptomatic and asymptomatic children. Statistical significance was determined using 95% confidence intervals.

### 2.7. Virus isolation

The stool sample was treated as previously described and inoculated on Vero cells (African green monkey kidney cells) obtained from ATCC (CCL-81) in a biosafety level 3 laboratory at the Pasteur Institute of Tunis (67). The inoculated cells were then maintained in Minimum Essential Medium (MEM) supplemented with 5% fetal bovine serum (FBS), incubated at 36°C and 5% CO2, and observed for cytopathic effect (CPE) for 7 days. In the absence of CPE, inoculated cells were harvested and clarified by centrifugation, and 200 μL of supernatant was inoculated onto a fresh cell culture monolayer and observed for an additional 7 days (53). Specimens were considered negative if no cytopathic effect was detected during 14 days after initial inoculation. For samples showing a cytopathic effect (CPE), the infected cells were then harvested and clarified by centrifugation. Virus suspensions were used for confirmation by SARS-CoV-2 real-time PCR.

## 3. Results

### 3.1. Positive SARS-CoV-2 RNA in nasopharyngeal swabs

All investigated cases presented an initial positive SARS-CoV-2 nasopharyngeal sample. Among them, 7 out of 71 (9.9%) children were asymptomatic, and 64 out of 71 (90.1%) were symptomatic.

**Figure 2:**
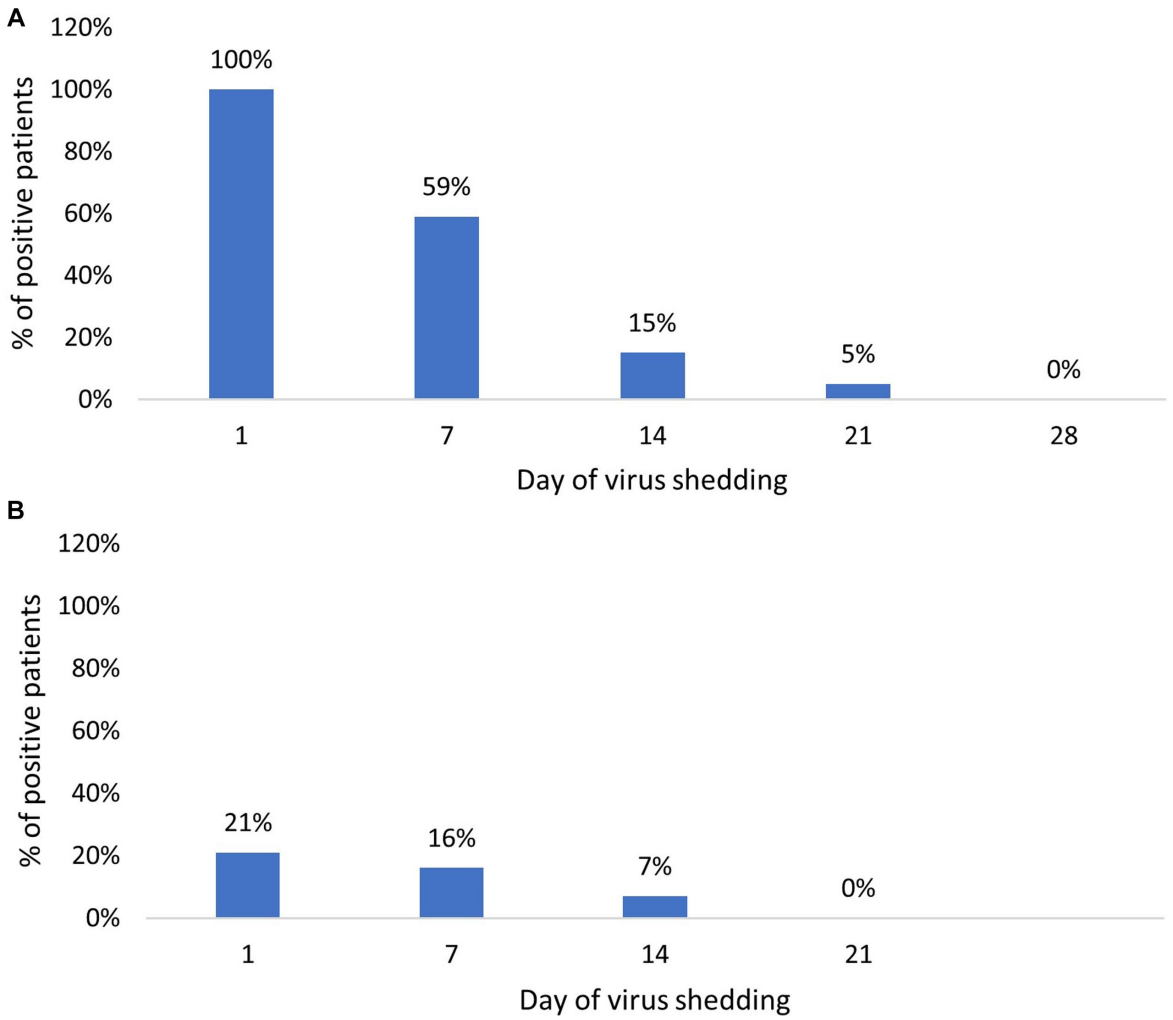
Percentage of SARS-CoV-2 positive patients over time: **(A)** Percentage of SARS-CoV-2 positive cases in nasopharyngeal samples; **(B)** Percentage of SARS-CoV-2 positive cases in stool samples.

They presented mild clinical signs, including fever (*n* = 46), cough (*n* = 43), headache (*n* = 32), loss of taste smell (*n* = 36), tiredness (*n* = 21), diarrhea (*n* = 32), vomiting (*n* = 16), muscle pain (*n* = 29), breathing difficulty (*n* = 4), conjunctivitis (*n* = 2), dyspnea (*n* = 3), urticaria (*n* = 2), and vertigo (*n* = 1). The duration of SARS-CoV-2 shedding in nasopharyngeal swabs varied between 7 and 21 days (average equal to 8.7 days). By days 7, 14, and 21, 42 (59%), 11 (15.5%), and 4 (5.6%) out of 71 children continued to shed SARS-CoV-2 RNA. All children stopped shedding SARS-CoV-2 RNA on day 28 (Figure 2A). The values of the threshold cycle (Ct values) varied from 13 to 34, 21 to 38, 22 to 37, and 28 to 35 on days 1, 7, 14, and 21, respectively. The median Ct values were 22, 27.3, 33.75, and 33.5 on days 1, 7, 14, and 21, respectively, as described in Table 2.

**Table 2:** Shedding duration and Ct values according to SARS-CoV-2 variant.

Patients	Variants	Clinical status Asymptomatic (A) Symptomatic (S)	Duration of nasopharyngeal shedding (days)	CT values				Duration of stool shedding (days)	CT values			
				Day 1	Day 7	Day 14	Day 21		Day 1	Day 7	Day 14	Day 21
P1	Alpha (B.1.1.7)	A	7	34-34^a^	33-35^a^	NEG^a^		7	25-28^a^	26-28^a^	NEG^a^	
P2		S	7	28-29^b^	32-35^a^	NEG^a^		7	30-32^a^	34-33^a^	NEG^a^	
P3		S	7	28-28^a^	32-31^a^	NEG^a^		14	30-30^a^	31-30^a^	32-30^b^	NEG^a^
P4	B.1.160	S	14	17-18^a^	28-26^a^	34-31^a^	NEG^a^	7	32-34^a^	36-36^a^	NEG^a^	
P5		A	7	29-30^a^	36-37^a^	NEG^a^		7	30-31^a^	32-31^a^	NEG^a^	
P6		S	7	24-24^b^	23-22^a^	NEG^a^		14	30-30^b^	31-31^a^	35-34^a^	NEG^a^
P7	Delta (AY.122)	S	14	24-25^b^	25-25^a^	23-22^a^	NEG^a^	14	31-33^a^	32-33^a^	34-32^b^	NEG^a^
P8		S	7	30-32^a^	27-26^a^	NEG^a^		7	32-31^a^	33-33^a^	NEG^b^	
P9		S	14	22-24^b^	31-30^a^	28-29^a^	NEG^a^	14	28-30^a^	30-30^a^	30-29^b^	NEG^a^
P10		S	7	20-24^b^	18-22^a^	NEG^a^		14	23-24^a^	24-24^a^	29-26^b^	NEG^a^
P11		S	7	25-28^b^	32-34^a^	NEG^a^		7	31-33^a^	35-34^a^	NEG^a^	
P12		S	7	23-26^a^	35-36^a^	NEG^a^		7	31-30^b^	34-34^a^	NEG^a^	
P13		S	7	28-28^b^	24-25^a^	NEG^a^		14	32-33^b^	33-33^b^	37-37^a^	NEG^a^
P14	OMICRON (BA.1.1.1)	S	7	22-21^b^	23-21^d^	NEG^a^		7	30-30^b^	33-31^b^	NEG^b^	
P15	ND	S	7	30-30^b^	34-32^b^	NEG		7	30-31^b^	32-36^b^	NEG	

^a^HKU protocol; ^b^IP2-IP4.

NEG, negative; ND, not determined.

### 3.2. Positive SARS-CoV-2 RNA in stool samples

Among the studied population, SARS-CoV-2 RNA was detected in stool samples of 15 out of 71 (21.1%) children. They are constituted by 13 out of 64 (20.3%) symptomatic and 2 out of 7 (28.6%) asymptomatic patients, with no statistically significant difference between the two groups (value of *p* > 0.05; x squared = 0.00042451, df = 1, value of *p* = 0.9836; Table 3).

**Table 3:** Prevalence of positive stool sample shedding cases among symptomatic and asymptomatic cases.

	Positive cases	Negative cases	Total cases	% of positive cases	Statistical test	ct value	Duration of virus shedding in stool samples
Symptomatic	13	51	64	20.3%	value of *p* = 0.9836	[23–37]	[7–14]
Asymptomatic	2	5	7	28.6%		[24–32]	7

A total of 36 positive samples were detected, with a threshold cycle (Ct) varying between 23 and 37 (Table 2). For samples obtained from the symptomatic group, the Ct values were between 23 and 37, while samples obtained from the asymptomatic group presented Ct values varying between 24 and 32 (Table 3).

The RNA-shedding duration was between 7 and 14 days. On days 7 and 14, 12 and 5 of 71 (16% and 7%) children remained positive by RT-PCR, respectively. No one of them continued to shed the RNA in stool samples on day 21 (Figure 2B). In symptomatic children, the duration of SARS-CoV-2 RNA excretion in stool samples varied between 7 and 14 days (average equivalent to 9.4), while in asymptomatic cases, the duration of viral RNA excretion was 7 days (Table 2).

### 3.3. SARS-CoV-2 variant identification

**Figure 3:**
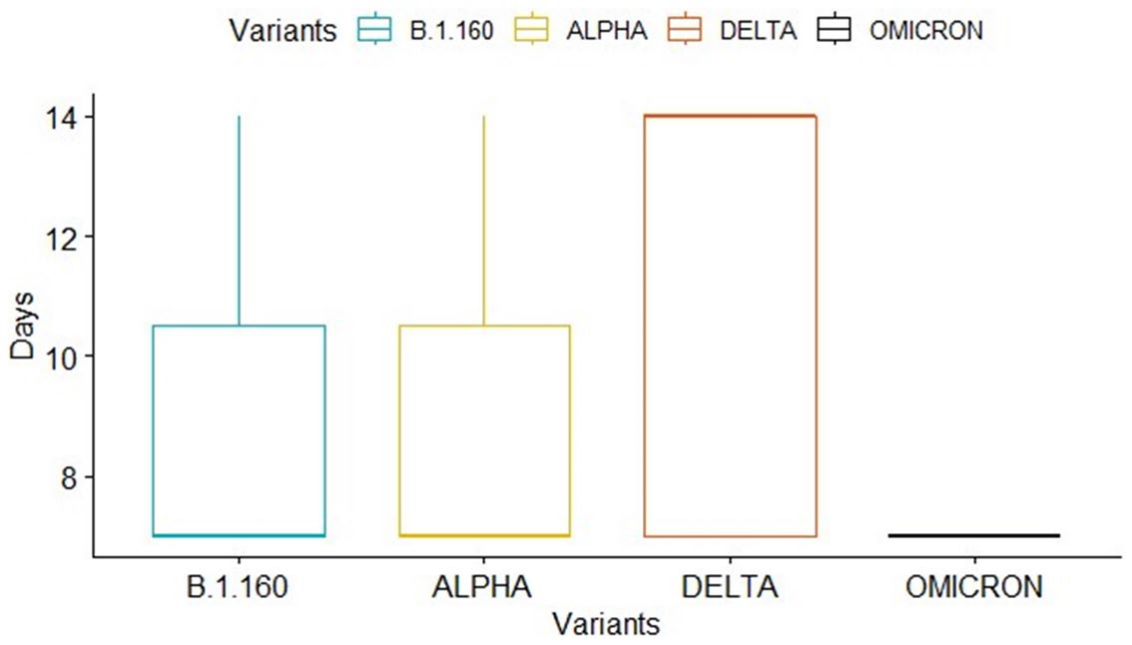
Presentation of SARS-CoV-2 RNA shedding duration for each variant using the boxplot package which describes data sets using 5 particular numbers: the minimum, first quartile, median, third quartile, and maximum. The box in the diagram begins with the first quartile and ends with the third quartile. Lines extend from the first quartile down to the minimum and from the third quartile up to the maximum.

With the aim of identifying variants of excreted viruses in stool samples, 14 full genome sequences were obtained from samples of 14 out of 15 children presenting fecal shedding (Table 2). For one child, sequences were not generated given the high real-time PCR Ct value. Four SARS-CoV-2 variants were detected: Alpha (B.1.1.7) (*n* = 3), B.1.160 (*n* = 3), Delta (AY.122 sub-variant) (*n* = 7), and Omicron (BA.1.1.1 sub-variant)(*n* = 1). The Delta variant was the most excreted variant (value of *p* < 0.05). The duration of excretion of positive fecal samples was variable among those variants (Figure 3). Patients infected with the Alpha and B.1.160 variants presented positive RNA results during 7 (*n* = 2) to 14 (*n* = 1) days of the infection, for each one. On the other hand, 7 children infected with the Delta variant (AY.122 sub-variant) presented positive results up to 14 days (*n* = 4) and 7 days (*n* = 3). For the Omicron variant (BA.1.1.1 sub-variant), the RNA shedding was limited to 7 days.

### 3.4. Virus isolation

Virus isolation using Vero cells showed non-specific modification of the cell aspect in positive stool samples. The observed modifications appeared between 4 and 7 days, respectively. After passaging, slight modifications appeared after 5 days. Specific SARS-CoV-2 RT-qPCR detection was negative for all obtained cell culture supernatants.

## 4. Discussion

Since the beginning of the COVID-19 pandemic, the fecal–oral excretion of infectious SARS-CoV-2 has been a matter of debate. Transmission via the fecal–oral route was previously demonstrated for other coronaviruses, such as MERS-CoV and SARS-CoV-1 (3, 68). Thus, in this study, we investigated the SARS-CoV-2 RNA shedding in stool samples obtained from symptomatic and asymptomatic COVID-19 pediatric cases infected with different virus variants between February 2021 and January 2022, along the Alpha, B.1.160, Delta, and Omicron waves in Tunisia (58, 69). To support the hypothesis of possible fecal–oral transmission, the virus viability in positive samples was investigated by inoculation with cell culture.

A proportion of 21.1% of infected children showed SARS-CoV-2 RNA shedding. The shedding occurred similarly among symptomatic and asymptomatic children. Previous studies showed that children with fecal excretion of viral RNA may be asymptomatic or present with clinical respiratory or gastrointestinal signs (19, 41, 42). The rate of positive RNA in fecal specimens of COVID-19 patients was controversial among the different published studies. An overview of the gastrointestinal shedding of SARS-CoV-2 in infected children suggested an average of 20–30% of positive fecal shedding in infants with and without gastrointestinal signs (38). However, other studies, mainly achieved rapidly at the beginning of the pandemic, reported 83.3 to 91.4% of RNA shedding in stool samples from children (3, 42, 70, 71). Other authors reported rates between 47 and 69% (72, 73).

Moreover, several data points regarding the shedding duration in stool samples suggest prolonged periods of up to 70 days or more (3, 19, 42, 74). The prolonged shedding period was mainly related to severe cases requiring hospitalization or immunocompromised patients (39, 75). Many studies reporting asymptomatic and moderate cases showed a shedding period between 18 and 32 days through the digestive tract (3, 72). In our series of investigations, the shedding period was at least 21 days in nasopharyngeal samples and at least 14 days in stool samples. Indeed, they present mild clinical forms with moderate respiratory and gastrointestinal signs.

In our study, the excreted viral RNA belonged to four variants of SARS-CoV-2: Alpha (B.1.1.7), B.1.160, Delta (B.1.617.2), and Omicron (B.1.1.529). The Delta variant (sub-variant AY.122) appears to be the most excreted, during the longest period, and with the highest viral load. In the literature, limited information was available about the rate of SARS-CoV-2 RNA shedding by children and adults according to the variant. Available data suggested increasing SARS-CoV-2 infection in the pediatric population, especially during the Delta variant wave, with higher transmissibility and pathogenicity than other variants (4, 10−14). It was suggested that the Delta variant is 60% more transmissible than the Alpha variant (32, 76). Furthermore, disease duration in children infected with the Delta variant was reported to be longer, in some cases exceeding 29 days (7, 77, 78).

It is worth noting that the detection of SARS-CoV-2 RNA in the framework of wastewater-based genomic surveillance is of great interest for tracking SARS-CoV-2 variants and the early management of new waves of the infection. It can complement clinical surveillance efforts and also offer more details about the evolutionary dynamics of SARS-CoV-2 (79).

To investigate the hypothesis of possible fecal–oral transmission, SARS-CoV-2 RNA detection and virus isolation on cell culture, followed by molecular confirmation, were used. Indeed, RNA shedding in stool samples could not reflect systematically the possible fecal–oral transmission, as the detection of SARS-CoV-2 RNA might be the result of virus replication into the gastrointestinal tract (38). In our study, a total of 36 samples were inoculated, and none of them showed any specific CPE until 14 days. Moreover, SARS-CoV-2 RNA detection in the cell culture supernatant was also negative. In this regard, the data in the literature are conflicting. Many studies have demonstrated the absence of cytopathic effect (CPE) in Vero cells after inoculation with stool samples (44, 45), while others have suggested the presence of infectious particles by the use of cell culture and electron microscopy (EM) visualization (48, 49). Reasonably, isolation in cell culture and observation of virus particles in EM are not sufficient to confirm the presence of viable particles. The use of more specific methods, such as molecular detection of sub-genomic RNA, is therefore highly recommended for investigating virus viability in infected biological specimens (80).

In our setting, the presence of CPE or cell modifications generated with stool samples could be the result of the multiplication of other enteric viruses, especially on Vero cells, which are permissive for the majority of cultured viruses. In this case, the initial evidence of SARS-CoV-2 RNA from the stool sample reflects only the presence of SARS-CoV-2 genomic RNA.

Indeed, in our study, investigated stool samples were all showing high CT values, evidence that makes virus isolation unlikely.

Nevertheless, the different parameters used for cell culture isolation may impact the sensitivity of virus detection. Furthermore, it is worth mentioning that our findings are derived from a relatively small number of positive stool samples. Conducting an analysis on a larger dataset of fecal specimens, if accessible, would support our findings. It is worth highlighting that during the study, a significant number of children, particularly adolescents, declined to provide stool samples.

From another point of view, it will be very interesting to investigate the potential correlations between fecal shedding patterns and variables such as the type of vaccine administered to participants and the severity of COVID-19 disease.

## 5. Conclusion

In conclusion, the exploration of SARS-CoV-2 shedding in stool samples bears significant relevance as it could contribute to a wider spread of the virus and environmental contamination. Our study has revealed that children can shed SARS-CoV-2 RNA in their nasopharyngeal and fecal samples for up to 21 and 14 days, respectively, particularly when infected with the Delta variant. However, none of the positive samples exhibited the presence of viable SARS-CoV-2 particles. Consequently, the likelihood of SARS-CoV-2 transmission via the fecal–oral route appears to be low. Further investigation involving a larger and more diverse population can provide additional support for our findings.

## Data availability statement

The datasets presented in this study can be found in online repositories. The names of the repository/repositories and accession number(s) can be found in the article/Supplementary material.

## Ethics statement

The studies involving humans were approved by the local Medical Ethics Committee of Bechir Hamza Children’s Hospital of Tunis, Tunisia, under the reference “12/2021.” The studies were conducted in accordance with the local legislation and institutional requirements. Written informed consent for participation in this study was provided by the participants’ legal guardians/next of kin.

## Author contributions

SH-B and HTr: conceptualization. HKh, MG, and SH-B: data curation. HKh, MG, SBH, HKr, HTo, AC, AS, ZM, NH, CC, MA, AP, and RA: investigation. HKh, MG, SBH, HKr, HTo, AC, AS, ZM, NH, CC, MA, AP, and RA: resources. HTr, SH-B, CC, AL, and AP: funding acquisition. HTr, SH-B, MA, and AP: supervision. HTr, AL, and SH-B: validation. HKh, HTr, AL, and SH-B: visualization, original draft preparation, and writing—review and editing. HKh and SH-B: writing. All authors reviewed the manuscript and agreed to its submission to this journal.

## Figures and Tables

**Table:** 

SARS-CoV-2	Severe acute respiratory syndrome coronavirus 2
GI	Gastrointestinal
RNA	Ribonucleic acid
RT-qPCR	Real-time quantitative reverse transcription polymerase chain reaction
ORF	Open reading frame
NGS	Next-generation sequencing
COVID-19	Coronavirus disease of 2019
VOCs	Variants of concern
ACE2	Angiotensin-converting enzyme 2
WHO	World Health Organization
PCR	Polymerase chain reaction
PBS	Polybutylene succinate
HKU	Hong Kong University
WHS	Whole-genome sequencing
BWA	Burrows-Wheeler Alignment
MEM	Minimum Essential Medium
FBS	Fetal bovine serum
CPE	Cytopathic effect
CT	Threshold cycle
EM	Electron microscopy

## Footnotes

^1^https://pangolin.cog-uk.io/

^2^https://clades.nextstrain.org/
