# Supplementary material for: SARS-CoV-2 excretion kinetics in nasopharyngeal and stool samples from the pediatric population
Source: Front Med (Lausanne). 2023 Oct 30;10:1226207. doi: 10.3389/fmed.2023.1226207 (PMC10643538; doi:10.3389/fmed.2023.1226207)
Supplement: Supplementary file 1 [file Table_1.DOCX]

**Supplementary Table 1:** Details of collected samples for each patient

| **Patients** | **Age (years)** | **Gender** | **Clinical signs** | **Nasopharyngeal samples** | | **Stool samples** | |
| --- | --- | --- | --- | --- | --- | --- | --- |
|  |  |  |  | **Number** | **Shedding duration(days)** | **Number** | **Shedding duration(days)** |
| p1 | 16 years | M | Symptomatic | 4 | 14 | 3 | 7 |
| p2 | 11 years | F | Symptomatic | 3 | 7 | 2 | 0 |
| p3 | 16 years | F | Symptomatic | 3 | 7 | 1 | 0 |
| p4 | 6 years | F | Asymptomatic | 3 | 7 | 2 | 7 |
| p5 | 15 years | M | Asymptomatic | 3 | 6 | 2 | 0 |
| p6 | 18 years | F | Symptomatic | 2 | 6 | 2 | 0 |
| p7 | 18 years | M | Symptomatic | 4 | 7 | 2 | 0 |
| p8 | 14 years | F | Symptomatic | 4 | 7 | 3 | 7 |
| p9 | 1-year et 8 months | M | Asymptomatic | 3 | 7 | 3 | 7 |
| p10 | 18 years | F | Symptomatic | 4 | 14 | 3 | 0 |
| p11 | 17 years | F | Asymptomatic | 3 | 6 | 2 | 0 |
| p12 | 15 years | F | Symptomatic | 3 | 10 | 1 | 0 |
| p13 | 14 years | M | Symptomatic | 2 | 5 | 1 | 0 |
| p14 | 16 years | M | Asymptomatic | 3 | 6 | 1 | 0 |
| p15 | 18 years | M | Symptomatic | 3 | 6 | 1 | 0 |
| p16 | 16 years | F | Symptomatic | 3 | 9 | 2 | 0 |
| p17 | 14 years | F | Symptomatic | 3 | 14 | 1 | 0 |
| p18 | 15 years | M | Symptomatic | 2 | 6 | 1 | 0 |
| p19 | 16 years | M | Symptomatic | 4 | 7 | 3 | 0 |
| p20 | 17 years | F | Symptomatic | 2 | 6 | 1 | 0 |
| p21 | 16 years | F | Symptomatic | 3 | 7 | 1 | 0 |
| p22 | 16 years | F | Symptomatic | 2 | 6 | 1 | 0 |
| p23 | 17 years | F | Symptomatic | 2 | 6 | 1 | 0 |
| p24 | 18 years | F | Symptomatic | 5 | 21 | 5 | 0 |
| p25 | 15 years | M | Asymptomatic | 2 | 6 | 1 | 0 |
| p26 | 18 years | M | Symptomatic | 2 | 6 | 1 | 0 |
| p27 | 16 years | F | Symptomatic | 2 | 6 | 1 | 0 |
| p28 | 16 years | F | Symptomatic | 4 | 14 | 3 | 7 |
| p29 | 17 years | F | Symptomatic | 4 | 7 | 3 | 14 |
| p30 | 15 years | M | Symptomatic | 3 | 7 | 1 | 0 |
| p31 | 15 years | F | Symptomatic | 4 | 14 | 3 | 0 |
| p32 | 13 years | F | Symptomatic | 3 | 7 | 1 | 8 |
| p33 | 18 years | M | Symptomatic | 3 | 7 | 2 |  |
| p34 | 18 years | F | Symptomatic | 4 | 14 | 1 | 14 |
| p35 | 17 years | M | Symptomatic | 2 | 6 | 1 | 0 |
| p36 | 12 years | F | Symptomatic | 2 | 7 | 1 | 0 |
| p37 | 12 years | M | Symptomatic | 2 | 6 | 2 | 14 |
| p38 | 0,58 | F | Symptomatic | 3 | 7 | 2 | 14 |
| p39 | 16 years | F | Symptomatic | 4 | 21 | 3 | 0 |
| p40 | 14 years | M | Symptomatic | 3 | 21 | 2 | 0 |
| p41 | 18 years | F | Symptomatic | 2 | 6 | 1 | 9 |
| p42 | 0,1 | M | Symptomatic | 5 | 21 | 3 | 0 |
| p43 | 18 years | F | Symptomatic | 3 | 14 | 3 | 0 |
| p44 | 10 years | M | Symptomatic | 2 | 7 | 1 | 0 |
| p45 | 11 years | F | Symptomatic | 2 | 7 | 1 | 7 |
| p46 | 19 years | F | Symptomatic | 1 | 6 | 1 | 7 |
| p47 | 18 years | F | Symptomatic | 3 | 7 | 1 | 7 |
| p48 | 16 years | F | Asymptomatic | 3 | 7 | 1 | 0 |
| p49 | 17 years | F | Symptomatic | 2 | 6 | 1 | 0 |
| p50 | 16 years | F | Symptomatic | 3 | 7 | 2 | 0 |
| p51 | 16 years | M | Symptomatic | 2 | 7 | 1 | 0 |
| p52 | 14 years | F | Symptomatic | 2 | 6 | 1 | 0 |
| p53 | 15 years | F | Symptomatic | 3 | 6 | 2 | 0 |
| p54 | 15 years | M | Symptomatic | 2 | 7 | 1 | 0 |
| p55 | 9 years | M | Symptomatic | 2 | 6 | 1 | 0 |
| p56 | 14 years | F | Symptomatic | 2 | 6 | 1 | 0 |
| p57 | 7 years | M | Symptomatic | 2 | 7 | 1 | 0 |
| p58 | 18 years | F | Symptomatic | 3 | 7 | 1 | 0 |
| p59 | 11 years | M | Symptomatic | 2 | 7 | 1 | 0 |
| p60 | 14 years | M | Symptomatic | 2 | 6 | 1 | 0 |
| p61 | 13 years | M | Symptomatic | 1 | 6 | 1 | 0 |
| p62 | 14 years | M | Symptomatic | 2 | 6 | 1 | 0 |
| p63 | 17 years | M | Symptomatic | 2 | 6 | 1 | 0 |
| p64 | 14 years | F | Symptomatic | 2 | 7 | 1 | 0 |
| p65 | 13 years | F | Symptomatic | 2 | 7 | 1 | 7 |
| p66 | 14 years | F | Symptomatic | 2 | 6 | 1 | 0 |
| p67 | 16 years | F | Symptomatic | 2 | 6 | 1 | 0 |
| p68 | 10 years | F | Symptomatic | 3 | 6 | 1 | 0 |
| p69 | 15 years | M | Symptomatic | 3 | 6 | 1 | 0 |
| p70 | 14 years | F | Symptomatic | 2 | 7 | 1 | 0 |
| p71 | 11 years | F | Symptomatic | 2 | 7 | 1 | 0 |
