# Supplementary material for: SARS-CoV-2 excretion kinetics in nasopharyngeal and stool samples from the pediatric population
Source: Front Med (Lausanne). 2023 Oct 30;10:1226207. doi: 10.3389/fmed.2023.1226207 (PMC10643538; doi:10.3389/fmed.2023.1226207)
Supplement: Supplementary file 2 [file Table_2.DOCX]

Supplementary material

The sequences generated in this work are available at GISAID database (https://www. gisaid.org) under the following accession numbers:

EPI_ISL_16847410

EPI_ISL_16847400

EPI_ISL_16847411

EPI_ISL_16847401

EPI_ISL_16847402

EPI_ISL_16847403

EPI_ISL_16847404

EPI_ISL_16847405

EPI_ISL_16847406

EPI_ISL_16847407

EPI_ISL_16847408

EPI_ISL_16847409

EPI_ISL_16854727

EPI_ISL_16854726
